# Supplementary figures and images for: Systematics and phylogeography of the Brazilian Atlantic Forest endemic harvestmen Neosadocus Mello-Leitão, 1926 (Arachnida: Opiliones: Gonyleptidae)
Source: PLoS One. 2021 Jun 2;16(6):e0249746. doi: 10.1371/journal.pone.0249746 (PMC8171921; doi:10.1371/journal.pone.0249746)

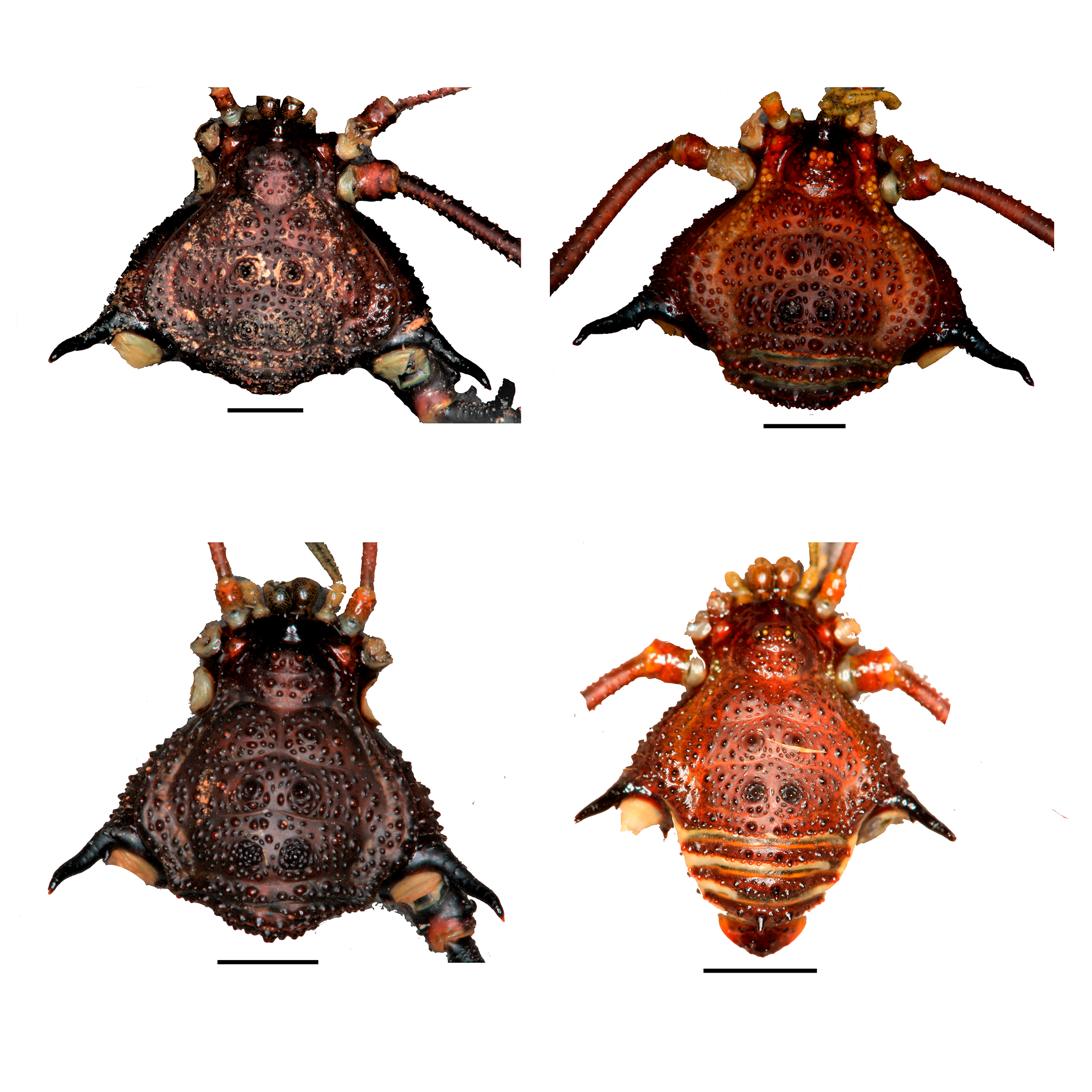

Supplement: S1 Fig — Scale bars: 1 mm. (TIF) [file pone.0249746.s001.tif]

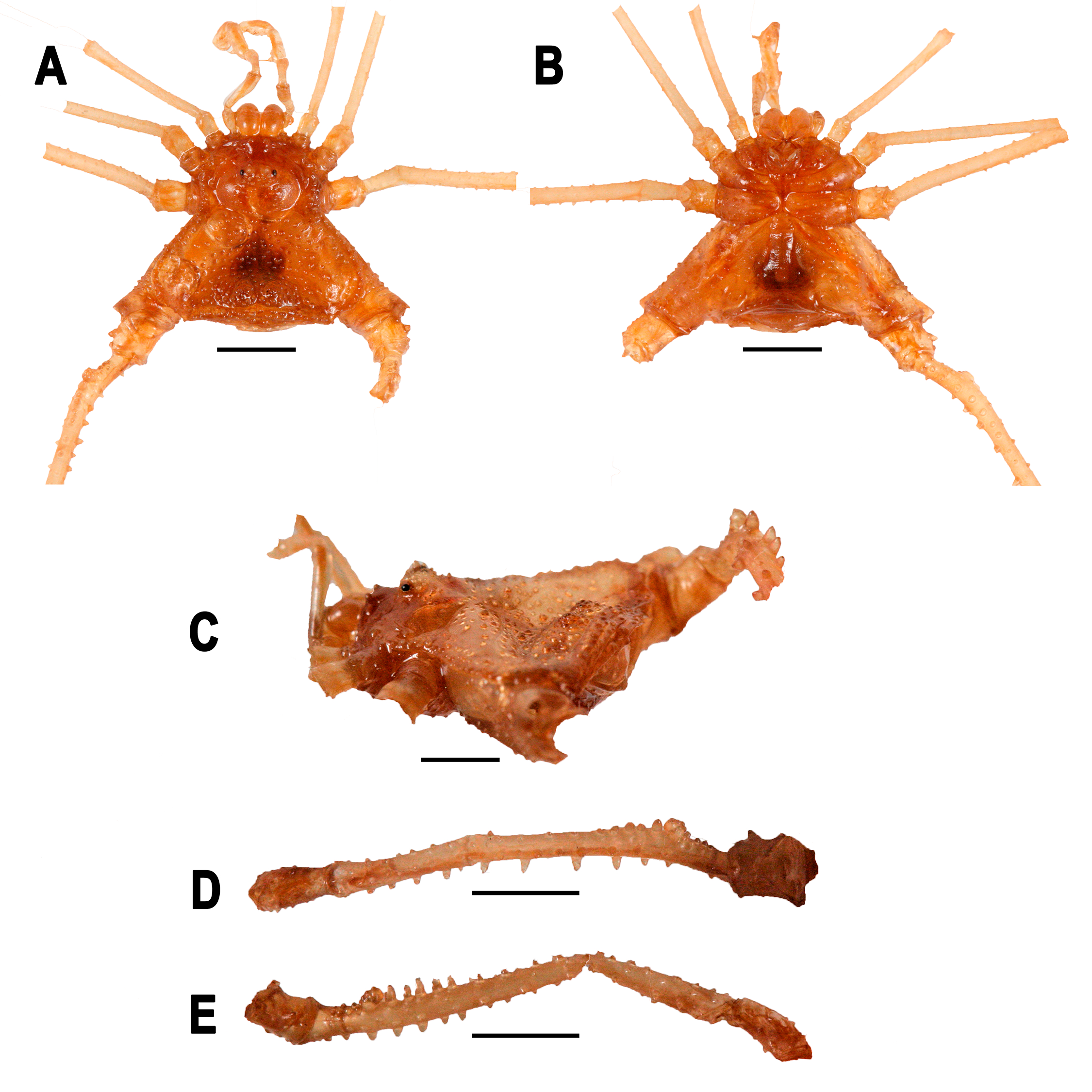

Supplement: S2 Fig — A. Dorsal view. B. Ventral view. C. Left lateral view. D–E. Right trochanter–patella IV (D: prolateral view; E: retrolateral view). Scale bars: 1 mm. (TIF) [file pone.0249746.s002.tif]

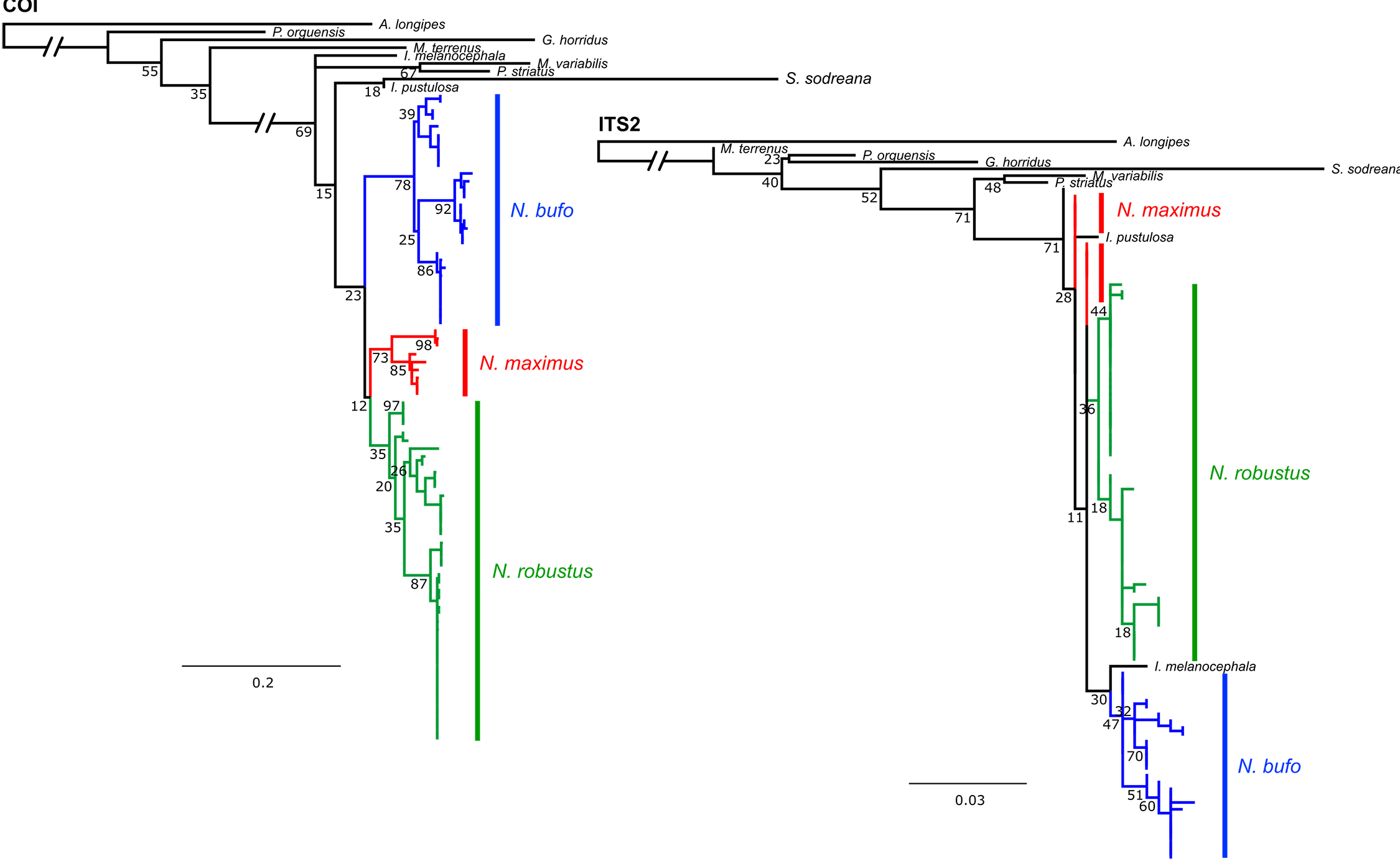

Supplement: S3 Fig — (TIF) [file pone.0249746.s003.tif]

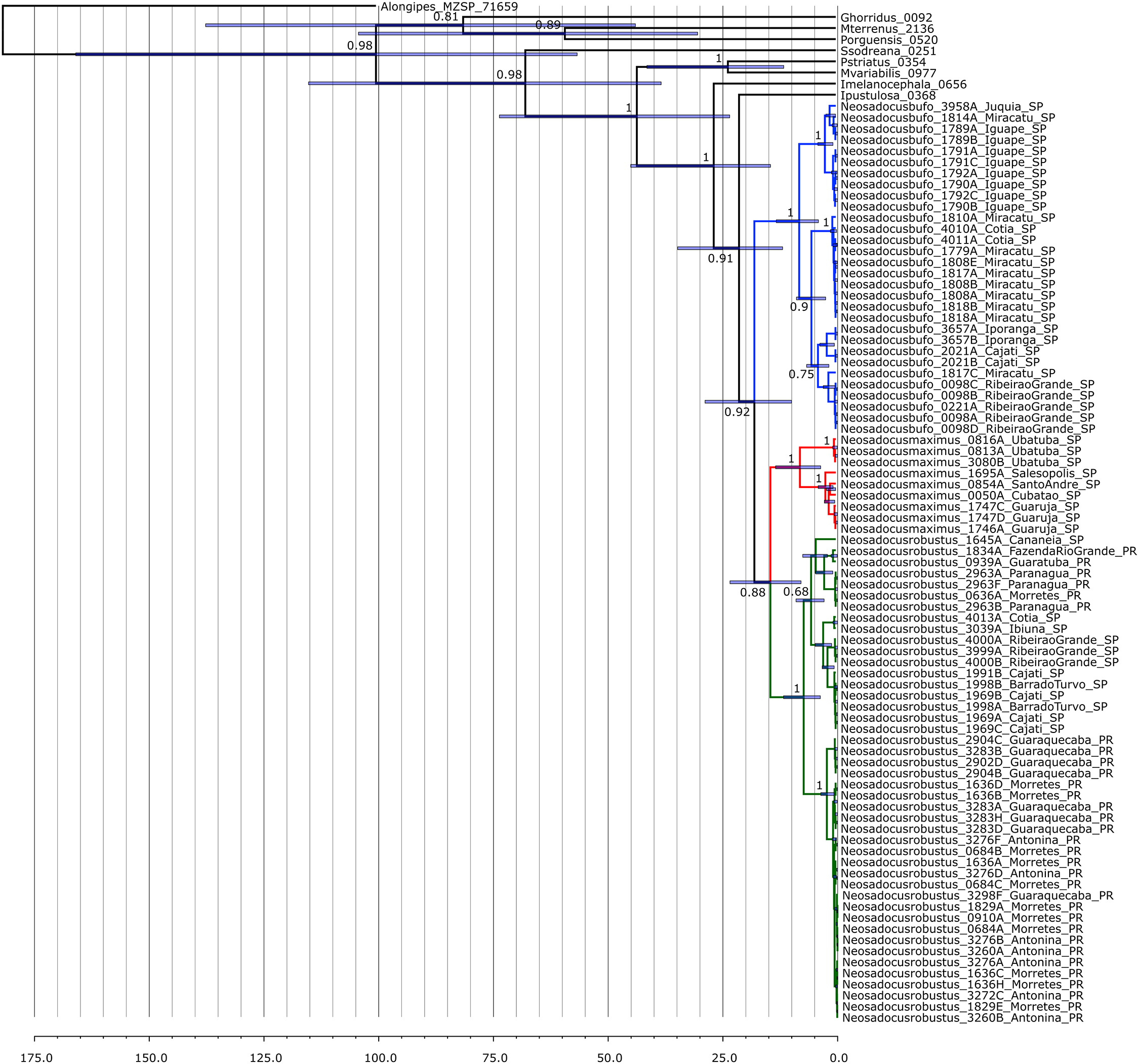

Supplement: S4 Fig — (TIF) [file pone.0249746.s004.tif]

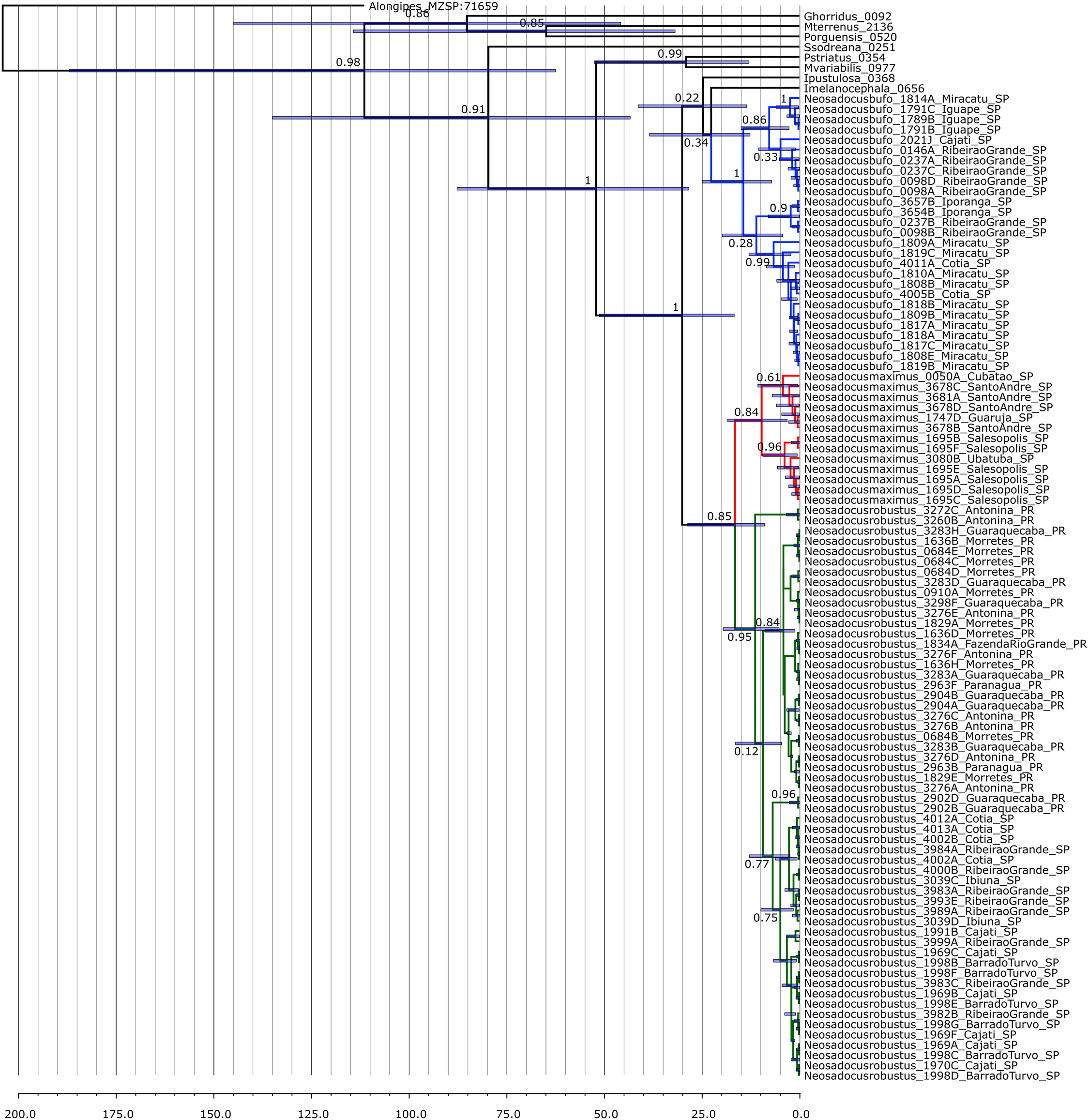

Supplement: S5 Fig — (TIF) [file pone.0249746.s005.tif]
