# Supplementary material for: Systematics and phylogeography of the Brazilian Atlantic Forest endemic harvestmen Neosadocus Mello-Leitão, 1926 (Arachnida: Opiliones: Gonyleptidae)
Source: PLoS One. 2021 Jun 2;16(6):e0249746. doi: 10.1371/journal.pone.0249746 (PMC8171921; doi:10.1371/journal.pone.0249746)
Supplement: S1 Table — (DOCX) [file pone.0249746.s006.docx]

**S1 Table.** Primers used for the amplification of each molecular marker.

| **Marker** | **Forward primer and sequence (5’ - 3’)** | **Reverse primer and sequence (5’ - 3’)** | **Reference** |
| --- | --- | --- | --- |
| **COI** | **dgLCO1490**  GGTCAACAAATCATAAAGAYATYGG | **dgHCO2198**  TAAACTTCAGGGTGACCAAARAAYCA | Meyer, 2003 |
| **ITS2** | **5.8SF**  CACGGGTCGATGAAGAACGC | **CAS28Sb1d**  TTCTTTTCCTCCSCTTAYTRATATGCTTAA | Ji *et al.*, 2003 |
