# Supplementary material for: Systematics and phylogeography of the Brazilian Atlantic Forest endemic harvestmen Neosadocus Mello-Leitão, 1926 (Arachnida: Opiliones: Gonyleptidae)
Source: PLoS One. 2021 Jun 2;16(6):e0249746. doi: 10.1371/journal.pone.0249746 (PMC8171921; doi:10.1371/journal.pone.0249746)
Supplement: S2 Table — (DOCX) [file pone.0249746.s007.docx]

S2 Table. COI and ITS2 haplotypes and BAPS results.

| Sample | Location | Hap_COI | BAPS_COI | Hap_ITS2 | BAPS_ITS2 |
| --- | --- | --- | --- | --- | --- |
| Neosadocusbufo_0098A | Ribeirão Grande-SP | H1 | B3 | H1 | B2 |
| Neosadocusbufo_0098B | Ribeirão Grande-SP | H1 | B3 | H2 | B2 |
| Neosadocusbufo_0098C | Ribeirão Grande-SP | H1 | B3 |  |  |
| Neosadocusbufo_0098D | Ribeirão Grande-SP | H1 | B3 | H3 | B2 |
| Neosadocusbufo_0146A | Ribeirão Grande-SP |  |  | H3 | B2 |
| Neosadocusbufo_0221A | Ribeirão Grande-SP | H1 | B3 |  |  |
| Neosadocusbufo_0237A | Ribeirão Grande-SP |  |  | H3 | B2 |
| Neosadocusbufo_0237B | Ribeirão Grande-SP |  |  | H2 | B2 |
| Neosadocusbufo_0237C | Ribeirão Grande-SP |  |  | H3 | B2 |
| Neosadocusbufo_1779A | Miracatu-SP | H2 | B4 |  |  |
| Neosadocusbufo_1808A | Miracatu-SP | H2 | B4 |  |  |
| Neosadocusbufo_1808B | Miracatu-SP | H2 | B4 | H6 | B1 |
| Neosadocusbufo_1808E | Miracatu-SP | H8 | B4 | H7 | B1 |
| Neosadocusbufo_1809A | Miracatu-SP |  |  | H8 | B1 |
| Neosadocusbufo_1809B | Miracatu-SP |  |  | H9 | B1 |
| Neosadocusbufo_1810A | Miracatu-SP | H9 | B4 | H9 | B1 |
| Neosadocusbufo_1814A | Miracatu-SP | H10 | B1 | H10 | B3 |
| Neosadocusbufo_1817A | Miracatu-SP | H2 | B4 | H9 | B1 |
| Neosadocusbufo_1817C | Miracatu-SP | H11 | B3 | H9 | B1 |
| Neosadocusbufo_1818A | Miracatu-SP | H2 | B4 | H9 | B1 |
| Neosadocusbufo_1818B | Miracatu-SP | H2 | B4 | H9 | B1 |
| Neosadocusbufo_1819B | Miracatu-SP |  |  | H9 | B1 |
| Neosadocusbufo_1819C | Miracatu-SP |  |  | H11 | B1 |
| Neosadocusbufo_2021A | Cajati-SP | H12 | B2 |  |  |
| Neosadocusbufo_2021B | Cajati-SP | H12 | B2 |  |  |
| Neosadocusbufo_2021J | Cajati-SP |  |  | H12 | B2 |
| Neosadocusbufo_1789A | Iguape-SP | H3 | B1 |  |  |
| Neosadocusbufo_1789B | Iguape-SP | H3 | B1 | H4 | B3 |
| Neosadocusbufo_1790A | Iguape-SP | H4 | B1 |  |  |
| Neosadocusbufo_1790B | Iguape-SP | H5 | B1 |  |  |
| Neosadocusbufo_1791A | Iguape-SP | H6 | B1 |  |  |
| Neosadocusbufo_1791B | Iguape-SP |  |  | H4 | B3 |
| Neosadocusbufo_1791C | Iguape-SP | H6 | B1 | H5 | B3 |
| Neosadocusbufo_1792A | Iguape-SP | H4 | B1 |  |  |
| Neosadocusbufo_1792C | Iguape-SP | H7 | B1 |  |  |
| Neosadocusbufo_3654B | Iporanga-SP |  |  | H2 | B2 |
| Neosadocusbufo_3657A | Iporanga-SP | H13 | B2 |  |  |
| Neosadocusbufo_3657B | Iporanga-SP | H13 | B2 | H2 | B2 |
| Neosadocusbufo_3958A | Juquiá-SP | H14 | B1 |  |  |
| Neosadocusbufo_4005B | Cotia-SP |  |  | H7 | B1 |
| Neosadocusbufo_4010A | Cotia-SP | H15 | B4 |  |  |
| Neosadocusbufo_4011A | Cotia-SP | H16 | B4 | H13 | B1 |
| Neosadocusmaximus_0050A | Cubatão-SP | H17 | M1 | H14 | M1 |
| Neosadocusmaximus_0854A | Santo André-SP | H20 | M1 |  |  |
| Neosadocusmaximus_3678B | Santo André-SP |  |  | H17 | M1 |
| Neosadocusmaximus_3678C | Santo André-SP |  |  | H17 | M1 |
| Neosadocusmaximus_3678D | Santo André-SP |  |  | H17 | M1 |
| Neosadocusmaximus_3681A | Santo André-SP |  |  | H17 | M1 |
| Neosadocusmaximus_1695A | Salesópolis-SP | H21 | M1 | H15 | M1 |
| Neosadocusmaximus_1695B | Salesópolis-SP |  |  | H16 | M1 |
| Neosadocusmaximus_1695C | Salesópolis-SP |  |  | H16 | M1 |
| Neosadocusmaximus_1695D | Salesópolis-SP |  |  | H16 | M1 |
| Neosadocusmaximus_1695E | Salesópolis-SP |  |  | H16 | M1 |
| Neosadocusmaximus_1695F | Salesópolis-SP |  |  | H16 | M1 |
| Neosadocusmaximus_1746A | Guarujá-SP | H22 | M1 |  |  |
| Neosadocusmaximus_1747C | Guarujá-SP | H23 | M1 |  |  |
| Neosadocusmaximus_1747D | Guarujá-SP | H22 | M1 | H17 | M1 |
| Neosadocusmaximus_0813A | Ubatuba-SP | H18 | M2 |  |  |
| Neosadocusmaximus_0816A | Ubatuba-SP | H19 | M2 |  |  |
| Neosadocusmaximus_3080B | Ubatuba-SP | H24 | M2 | H18 | M2 |
| Neosadocusrobustus_3982B | Ribeirão Grande-SP |  |  | H28 | R2 |
| Neosadocusrobustus_3983A | Ribeirão Grande-SP |  |  | H29 | R2 |
| Neosadocusrobustus_3983C | Ribeirão Grande-SP |  |  | H28 | R2 |
| Neosadocusrobustus_3984A | Ribeirão Grande-SP |  |  | H30 | R3 |
| Neosadocusrobustus_3989A | Ribeirão Grande-SP |  |  | H31 | R2 |
| Neosadocusrobustus_3993E | Ribeirão Grande-SP |  |  | H29 | R2 |
| Neosadocusrobustus_3999A | Ribeirão Grande-SP | H40 | R3 | H32 | R2 |
| Neosadocusrobustus_4000A | Ribeirão Grande-SP | H41 | R3 |  |  |
| Neosadocusrobustus_4000B | Ribeirão Grande-SP | H40 | R3 | H29 | R2 |
| Neosadocusrobustus_1969A | Cajati-SP | H32 | R4 | H21 | R2 |
| Neosadocusrobustus_1969B | Cajati-SP | H32 | R4 | H21 | R2 |
| Neosadocusrobustus_1969C | Cajati-SP | H32 | R4 | H21 | R2 |
| Neosadocusrobustus_1969F | Cajati-SP |  |  | H21 | R2 |
| Neosadocusrobustus_1970C | Cajati-SP |  |  | H21 | R2 |
| Neosadocusrobustus_1991B | Cajati-SP | H33 | R4 | H22 | R2 |
| Neosadocusrobustus_4002A | Cotia-SP |  |  | H30 | R3 |
| Neosadocusrobustus_4002B | Cotia-SP |  |  | H30 | R3 |
| Neosadocusrobustus_4012A | Cotia-SP |  |  | H30 | R3 |
| Neosadocusrobustus_4013A | Cotia-SP | H42 | R3 | H30 | R3 |
| Neosadocusrobustus_0636A | Morretes-PR | H25 | R6 |  |  |
| Neosadocusrobustus_0684A | Morretes-PR | H26 | R2 |  |  |
| Neosadocusrobustus_0684B | Morretes-PR | H26 | R2 | H19 | R1 |
| Neosadocusrobustus_0684C | Morretes-PR | H26 | R2 | H19 | R1 |
| Neosadocusrobustus_0684D | Morretes-PR |  |  | H19 | R1 |
| Neosadocusrobustus_0684E | Morretes-PR |  |  | H19 | R1 |
| Neosadocusrobustus_0910A | Morretes-PR | H27 | R2 | H19 | R1 |
| Neosadocusrobustus_1636A | Morretes-PR | H26 | R2 |  |  |
| Neosadocusrobustus_1636B | Morretes-PR | H29 | R2 | H20 | R1 |
| Neosadocusrobustus_1636C | Morretes-PR | H26 | R2 |  |  |
| Neosadocusrobustus_1636D | Morretes-PR | H29 | R2 | H20 | R1 |
| Neosadocusrobustus_1636H | Morretes-PR | H26 | R2 | H20 | R1 |
| Neosadocusrobustus_1829A | Morretes-PR | H27 | R2 | H19 | R1 |
| Neosadocusrobustus_1829E | Morretes-PR | H26 | R2 | H19 | R1 |
| Neosadocusrobustus_2902B | Guaraqueçaba-PR |  |  | H23 | R1 |
| Neosadocusrobustus_2902D | Guaraqueçaba-PR | H34 | R1 | H23 | R1 |
| Neosadocusrobustus_2904A | Guaraqueçaba-PR |  |  | H24 | R1 |
| Neosadocusrobustus_2904B | Guaraqueçaba-PR | H34 | R1 | H24 | R1 |
| Neosadocusrobustus_2904C | Guaraqueçaba-PR | H35 | R1 |  |  |
| Neosadocusrobustus_3283A | Guaraqueçaba-PR | H37 | R2 | H20 | R1 |
| Neosadocusrobustus_3283B | Guaraqueçaba-PR | H34 | R1 | H20 | R1 |
| Neosadocusrobustus_3283D | Guaraqueçaba-PR | H38 | R2 | H27 | R1 |
| Neosadocusrobustus_3283H | Guaraqueçaba-PR | H38 | R2 | H20 | R1 |
| Neosadocusrobustus_3298F | Guaraqueçaba-PR | H39 | R2 | H20 | R1 |
| Neosadocusrobustus_3260A | Antonina-PR | H26 | R2 |  |  |
| Neosadocusrobustus_3260B | Antonina-PR | H26 | R2 | H26 | R1 |
| Neosadocusrobustus_3272C | Antonina-PR | H26 | R2 | H26 | R1 |
| Neosadocusrobustus_3276A | Antonina-PR | H26 | R2 | H19 | R1 |
| Neosadocusrobustus_3276B | Antonina-PR | H26 | R2 | H19 | R1 |
| Neosadocusrobustus_3276C | Antonina-PR |  |  | H19 | R1 |
| Neosadocusrobustus_3276D | Antonina-PR | H26 | R2 | H19 | R1 |
| Neosadocusrobustus_3276E | Antonina-PR |  |  | H19 | R1 |
| Neosadocusrobustus_3276F | Antonina-PR | H26 | R2 | H19 | R1 |
| Neosadocusrobustus_1645A | Cananéia-SP | H30 | R5 |  |  |
| Neosadocusrobustus_1998A | Barra do Turvo-SP | H31 | R4 |  |  |
| Neosadocusrobustus_1998B | Barra do Turvo-SP | H31 | R4 | H21 | R2 |
| Neosadocusrobustus_1998C | Barra do Turvo-SP |  |  | H21 | R2 |
| Neosadocusrobustus_1998D | Barra do Turvo-SP |  |  | H21 | R2 |
| Neosadocusrobustus_1998E | Barra do Turvo-SP |  |  | H21 | R2 |
| Neosadocusrobustus_1998F | Barra do Turvo-SP |  |  | H21 | R2 |
| Neosadocusrobustus_1998G | Barra do Turvo-SP |  |  | H21 | R2 |
| Neosadocusrobustus_3039A | Ibiúna-SP | H36 | R3 |  |  |
| Neosadocusrobustus_3039C | Ibiúna-SP |  |  | H25 | R2 |
| Neosadocusrobustus_3039D | Ibiúna-SP |  |  | H25 | R2 |
| Neosadocusrobustus_0939A | Guaratuba-PR | H28 | R6 |  |  |
| Neosadocusrobustus_1834A | Faz Rio Grande-PR | H31 | R6 | H20 | R1 |
| Neosadocusrobustus_2963A | Paranaguá-PR | H25 | R6 |  |  |
| Neosadocusrobustus_2963B | Paranaguá-PR | H25 | R6 | H20 | R1 |
| Neosadocusrobustus_2963F | Paranaguá-PR | H25 | R6 | H20 | R1 |
