# Supplementary material for: Systematics and phylogeography of the Brazilian Atlantic Forest endemic harvestmen Neosadocus Mello-Leitão, 1926 (Arachnida: Opiliones: Gonyleptidae)
Source: PLoS One. 2021 Jun 2;16(6):e0249746. doi: 10.1371/journal.pone.0249746 (PMC8171921; doi:10.1371/journal.pone.0249746)
Supplement: S3 Table — (DOCX) [file pone.0249746.s008.docx]

**S3 Table.** Genetic distances between *Neosadocus* species obtained for **COI** sequences. Above diagonal, the average number of sequences’ pairwise differences (D); below diagonal, the corrected average number of pairwise differences (D_A_). In gray, the average number of differences within species.

|  | ***N_bufo*** | ***N_maximus*** | ***N_robustus*** |
| --- | --- | --- | --- |
| ***N_bufo*** | 21.17 | 46.91 | 46.17 |
| ***N_maximus*** | 26.63 | 19.39 | 39.59 |
| ***N_robustus*** | 27.12 | 21.43 | 16.94 |
