# Supplementary material for: Systematics and phylogeography of the Brazilian Atlantic Forest endemic harvestmen Neosadocus Mello-Leitão, 1926 (Arachnida: Opiliones: Gonyleptidae)
Source: PLoS One. 2021 Jun 2;16(6):e0249746. doi: 10.1371/journal.pone.0249746 (PMC8171921; doi:10.1371/journal.pone.0249746)
Supplement: S4 Table — (DOCX) [file pone.0249746.s009.docx]

**S4 Table.** Genetic distances between *Neosadocus* species obtained for **ITS2** sequences. Above diagonal, the average number of sequences’ pairwise differences (D); below diagonal, the corrected average number of pairwise differences (D_A_). In gray, the average number of differences within species.

|  | ***N_bufo*** | ***N_maximus*** | ***N_robustus*** |
| --- | --- | --- | --- |
| ***N_bufo*** | 3.869 | 7.0912 | 8.007 |
| ***N_maximus*** | 4.5413 | 1.2308 | 4.022 |
| ***N_robustus*** | 4.5994 | 1.9339 | 2.945 |
