# Supplementary material for: Systematics and phylogeography of the Brazilian Atlantic Forest endemic harvestmen Neosadocus Mello-Leitão, 1926 (Arachnida: Opiliones: Gonyleptidae)
Source: PLoS One. 2021 Jun 2;16(6):e0249746. doi: 10.1371/journal.pone.0249746 (PMC8171921; doi:10.1371/journal.pone.0249746)
Supplement: S5 Table — Above diagonal, the average number of sequences’ pairwise differences (D); below diagonal, the corrected average number of pairwise differences (DA). In gray, the average number of differences within populations. (DOCX) [file pone.0249746.s010.docx]

**S5 Table.** Genetic distances between ***N. bufo*** populations obtained for **COI** sequences. Above diagonal, the average number of sequences’ pairwise differences (D); below diagonal, the corrected average number of pairwise differences (D_A_). In gray, the average number of differences within populations.

|  | **N_bufo_Ribeirao_Grande** | **N_bufo_Miracatu** | **N_bufo_Cajati** | **N_bufo_Iguape** | **N_bufo_Iporanga** | **N_bufo_Juquia** | **N_bufo_Cotia** |
| --- | --- | --- | --- | --- | --- | --- | --- |
| **N_bufo_Ribeirao_Grande** | 0.000 | 22.000 | 17.000 | 32.000 | 20.000 | 33.000 | 24.000 |
| **N_bufo_Miracatu** | 16.667 | 10.667 | 19.600 | 25.600 | 25.000 | 28.000 | 7.600 |
| **N_bufo_Cajati** | 17.000 | 14.267 | 0.000 | 28.500 | 11.000 | 28.000 | 21.000 |
| **N_bufo_Iguape** | 29.536 | 17.802 | 26.036 | 4.929 | 27.500 | 12.000 | 29.000 |
| **N_bufo_Iporanga** | 20.000 | 19.667 | 11.000 | 25.036 | 0.000 | 27.000 | 28.000 |
| **N_bufo_Juquia** | 33.000 | 23.467 | 28.000 | 9.536 | 27.000 | 0.000 | 33.000 |
| **N_bufo_Cotia** | 22.000 | 0.267 | 19.000 | 24.536 | 26.000 | 31.000 | 4.000 |
