# Supplementary material for: Systematics and phylogeography of the Brazilian Atlantic Forest endemic harvestmen Neosadocus Mello-Leitão, 1926 (Arachnida: Opiliones: Gonyleptidae)
Source: PLoS One. 2021 Jun 2;16(6):e0249746. doi: 10.1371/journal.pone.0249746 (PMC8171921; doi:10.1371/journal.pone.0249746)
Supplement: S6 Table — Above diagonal, the average number of sequences’ pairwise differences (D); below diagonal, the corrected average number of pairwise differences (DA). In gray, the average number of differences within populations. (DOCX) [file pone.0249746.s011.docx]

**S6 Table.** Genetic distances between ***N. bufo*** populations obtained for **ITS2** sequences. Above diagonal, the average number of sequences’ pairwise differences (D); below diagonal, the corrected average number of pairwise differences (D_A_). In gray, the average number of differences within populations.

|  | **N_bufo_Ribeirao_Grande** | **N_bufo_Miracatu** | **N_bufo_Cajati** | **N_bufo_Iguape** | **N_bufo_Iporanga** | **N_bufo_Cotia** |
| --- | --- | --- | --- | --- | --- | --- |
| **N_bufo_Ribeirao_Grande** | 1.238 | 5.321 | 2.143 | 4.810 | 1.571 | 7.286 |
| **N_bufo_Miracatu** | 3.695 | 2.015 | 3.917 | 5.028 | 3.917 | 2.750 |
| **N_bufo_Cajati** | 1.524 | 2.909 | 0.000 | 2.667 | 2.000 | 6.000 |
| **N_bufo_Iguape** | 3.857 | 3.687 | 2.333 | 0.667 | 4.667 | 7.333 |
| **N_bufo_Iporanga** | 0.952 | 2.909 | 2.000 | 4.333 | 0.000 | 6.000 |
| **N_bufo_Cotia** | 5.667 | 0.742 | 5.000 | 6.000 | 5.000 | 2.000 |
