# Supplementary material for: Systematics and phylogeography of the Brazilian Atlantic Forest endemic harvestmen Neosadocus Mello-Leitão, 1926 (Arachnida: Opiliones: Gonyleptidae)
Source: PLoS One. 2021 Jun 2;16(6):e0249746. doi: 10.1371/journal.pone.0249746 (PMC8171921; doi:10.1371/journal.pone.0249746)
Supplement: S7 Table — Above diagonal, the average number of sequences’ pairwise differences (D); below diagonal, the corrected average number of pairwise differences (DA). In gray, the average number of differences within populations. (DOCX) [file pone.0249746.s012.docx]

**S7 Table.** Genetic distances between ***N. maximus*** populations obtained for **COI** sequences. Above diagonal, the average number of sequences’ pairwise differences (D); below diagonal, the corrected average number of pairwise differences (D_A_). In gray, the average number of differences within populations.

|  | **N_maximus_Cubatao** | **N_maximus_Santo_Andre** | **N_maximus_Salesopolis** | **N_maximus_Guaruja** | **N_maximus_Ubatuba** |
| --- | --- | --- | --- | --- | --- |
| **N_maximus_Cubatao** | 0.000 | 8.000 | 12.000 | 7.333 | 33.000 |
| **N_maximus_Santo_Andre** | 8.000 | 0.000 | 13.000 | 8.333 | 32.000 |
| **N_maximus_Salesopolis** | 12.000 | 13.000 | 0.000 | 11.333 | 30.667 |
| **N_maximus_Guaruja** | 7.000 | 8.000 | 11.000 | 0.667 | 32.333 |
| **N_maximus_Ubatuba** | 32.333 | 31.333 | 30.000 | 31.333 | 1.333 |
