# Supplementary material for: Systematics and phylogeography of the Brazilian Atlantic Forest endemic harvestmen Neosadocus Mello-Leitão, 1926 (Arachnida: Opiliones: Gonyleptidae)
Source: PLoS One. 2021 Jun 2;16(6):e0249746. doi: 10.1371/journal.pone.0249746 (PMC8171921; doi:10.1371/journal.pone.0249746)
Supplement: S8 Table — Above diagonal, the average number of sequences’ pairwise differences (D); below diagonal, the corrected average number of pairwise differences (DA). In gray, the average number of differences within populations. (DOCX) [file pone.0249746.s013.docx]

**S8 Table.** Genetic distances between ***N. maximus*** populations obtained for **ITS2** sequences. Above diagonal, the average number of sequences’ pairwise differences (D); below diagonal, the corrected average number of pairwise differences (D_A_). In gray, the average number of differences within populations.

|  | **N_maximus_Cubatao** | **N_maximus_Santo_Andre** | **N_maximus_Salesopolis** | **N_maximus_Guaruja** | **N_maximus_Ubatuba** |
| --- | --- | --- | --- | --- | --- |
| **N_maximus_Cubatao** | 0.000 | 1.000 | 1.167 | 1.000 | 2.000 |
| **N_maximus_Santo_Andre** | 1.000 | 0.000 | 1.833 | 0.000 | 3.000 |
| **N_maximus_Salesopolis** | 1.000 | 1.667 | 0.333 | 1.833 | 1.167 |
| **N_maximus_Guaruja** | 1.000 | 0.000 | 1.667 | 0.000 | 3.000 |
| **N_maximus_Ubatuba** | 2.000 | 3.000 | 1.000 | 3.000 | 0.000 |
