# Supplementary material for: Systematics and phylogeography of the Brazilian Atlantic Forest endemic harvestmen Neosadocus Mello-Leitão, 1926 (Arachnida: Opiliones: Gonyleptidae)
Source: PLoS One. 2021 Jun 2;16(6):e0249746. doi: 10.1371/journal.pone.0249746 (PMC8171921; doi:10.1371/journal.pone.0249746)
Supplement: S9 Table — Above diagonal, the average number of sequences’ pairwise differences (D); below diagonal, the corrected average number of pairwise differences (DA). In gray, the average number of differences within populations. (DOCX) [file pone.0249746.s014.docx]

**S9 Table.** Genetic distances between ***N. robustus*** populations obtained for **COI** sequences. Above diagonal, the average number of sequences’ pairwise differences (D); below diagonal, the corrected average number of pairwise differences (D_A_). In gray, the average number of differences within populations.

|  | **N_robustus_Ribeirao_Grande** | **N_robustus_Cajati** | **N_robustus_Cotia** | **N_robustus_Morretes** | **N_robustus_Guaraquecaba** | **N_robustus_Antonina** | **N_robustus_Cananeia** | **N_robustus_Barra_do_Turvo** | **N_robustus_Ibiuna** | **N_robustus_Guaratuba** | **N_robustus_Faz_Rio_Grande** | **N_robustus_Paranagua** |
| --- | --- | --- | --- | --- | --- | --- | --- | --- | --- | --- | --- | --- |
| **N_robustus_Ribeirao_Grande** | 0.667 | 13.833 | 14.333 | 26.333 | 28.458 | 26.333 | 26.333 | 13.333 | 12.333 | 21.333 | 23.333 | 26.333 |
| **N_robustus_Cajati** | 13.000 | 1.000 | 16.500 | 23.667 | 25.125 | 23.500 | 27.500 | 0.500 | 14.500 | 22.500 | 24.500 | 25.500 |
| **N_robustus_Cotia** | 14.000 | 16.000 | 0.000 | 25.667 | 27.125 | 26.000 | 22.000 | 16.000 | 2.000 | 21.000 | 23.000 | 22.000 |
| **N_robustus_Morretes** | 23.394 | 20.561 | 23.061 | 5.212 | 7.990 | 2.667 | 30.667 | 23.167 | 23.667 | 22.667 | 26.000 | 25.667 |
| **N_robustus_Guaraquecaba** | 24.679 | 21.179 | 23.679 | 1.937 | 6.893 | 5.625 | 31.000 | 24.625 | 25.125 | 24.000 | 27.750 | 30.000 |
| **N_robustus_Antonina** | 26.000 | 23.000 | 26.000 | 0.061 | 2.179 | 0.000 | 31.000 | 23.000 | 24.000 | 23.000 | 27.000 | 28.000 |
| **N_robustus_Cananeia** | 26.000 | 27.000 | 22.000 | 28.061 | 27.554 | 31.000 | 0.000 | 27.000 | 20.000 | 26.000 | 26.000 | 27.000 |
| **N_robustus_Barra_do_Turvo** | 13.000 | 0.000 | 16.000 | 20.561 | 21.179 | 23.000 | 27.000 | 0.000 | 14.000 | 22.000 | 24.000 | 25.000 |
| **N_robustus_Ibiuna** | 12.000 | 14.000 | 2.000 | 21.061 | 21.679 | 24.000 | 20.000 | 14.000 | 0.000 | 19.000 | 21.000 | 20.000 |
| **N_robustus_Guaratuba** | 21.000 | 22.000 | 21.000 | 20.061 | 20.554 | 23.000 | 26.000 | 22.000 | 19.000 | 0.000 | 4.000 | 15.000 |
| **N_robustus_Faz_Rio_Grande** | 23.000 | 24.000 | 23.000 | 23.394 | 24.304 | 27.000 | 26.000 | 24.000 | 21.000 | 4.000 | 0.000 | 15.000 |
| **N_robustus_Paranagua** | 26.000 | 25.000 | 22.000 | 23.061 | 26.554 | 28.000 | 27.000 | 25.000 | 20.000 | 15.000 | 15.000 | 0.000 |
