# Supplementary material for: Systematics and phylogeography of the Brazilian Atlantic Forest endemic harvestmen Neosadocus Mello-Leitão, 1926 (Arachnida: Opiliones: Gonyleptidae)
Source: PLoS One. 2021 Jun 2;16(6):e0249746. doi: 10.1371/journal.pone.0249746 (PMC8171921; doi:10.1371/journal.pone.0249746)
Supplement: S10 Table — Above diagonal, the average number of sequences’ pairwise differences (D); below diagonal, the corrected average number of pairwise differences (DA). In gray, the average number of differences within populations. (DOCX) [file pone.0249746.s015.docx]

**S10 Table.** Genetic distances between ***N. robustus*** populations obtained for **ITS2** sequences. Above diagonal, the average number of sequences’ pairwise differences (D); below diagonal, the corrected average number of pairwise differences (D_A_). In gray, the average number of differences within populations.

|  | **N_robustus_Ribeirao_Grande** | **N_robustus_Cajati** | **N_robustus_Cotia** | **N_robustus_Morretes** | **N_robustus_Guaraquecaba** | **N_robustus_Antonina** | **N_robustus_Barra_do_Turvo** | **N_robustus_Ibiuna** | **N_robustus_Faz_Rio_Grande** | **N_robustus_Paranagua** |
| --- | --- | --- | --- | --- | --- | --- | --- | --- | --- | --- |
| **N_robustus_Ribeirao_Grande** | 1.714 | 2.396 | 3.250 | 4.950 | 3.917 | 4.750 | 2.250 | 2.000 | 4.250 | 4.250 |
| **N_robustus_Cajati** | 1.372 | 0.333 | 5.000 | 3.467 | 2.944 | 3.167 | 0.167 | 1.167 | 4.167 | 4.167 |
| **N_robustus_Cotia** | 2.393 | 4.833 | 0.000 | 7.700 | 6.667 | 7.500 | 5.000 | 4.000 | 7.000 | 7.000 |
| **N_robustus_Morretes** | 3.860 | 3.067 | 7.467 | 0.467 | 1.300 | 0.650 | 3.300 | 4.300 | 0.700 | 0.700 |
| **N_robustus_Guaraquecaba** | 2.198 | 1.917 | 5.806 | 0.206 | 1.722 | 1.444 | 2.778 | 3.778 | 1.222 | 1.222 |
| **N_robustus_Antonina** | 3.464 | 2.571 | 7.071 | -0.012 | 0.155 | 0.857 | 3.000 | 4.000 | 1.000 | 1.000 |
| **N_robustus_Barra_do_Turvo** | 1.393 | 0.000 | 5.000 | 3.067 | 1.917 | 2.571 | 0.000 | 1.000 | 4.000 | 4.000 |
| **N_robustus_Ibiuna** | 1.143 | 1.000 | 4.000 | 4.067 | 2.917 | 3.571 | 1.000 | 0.000 | 5.000 | 5.000 |
| **N_robustus_Faz_Rio_Grande** | 3.393 | 4.000 | 7.000 | 0.467 | 0.361 | 0.571 | 4.000 | 5.000 | 0.000 | 0.000 |
| **N_robustus_Paranagua** | 3.393 | 4.000 | 7.000 | 0.467 | 0.361 | 0.571 | 4.000 | 5.000 | 0.000 | 0.000 |
