# Supplementary material for: Systematics and phylogeography of the Brazilian Atlantic Forest endemic harvestmen Neosadocus Mello-Leitão, 1926 (Arachnida: Opiliones: Gonyleptidae)
Source: PLoS One. 2021 Jun 2;16(6):e0249746. doi: 10.1371/journal.pone.0249746 (PMC8171921; doi:10.1371/journal.pone.0249746)
Supplement: S11 Table — (DOCX) [file pone.0249746.s016.docx]

**S11 Table.** Pairwise Φ_ST_ values between ***N. bufo*** populations obtained for **COI** sequences (*p<0.05).

|  | **N_bufo_Ribeirao_Grande** | **N_bufo_Miracatu** | **N_bufo_Cajati** | **N_bufo_Iguape** | **N_bufo_Iporanga** | **N_bufo_Juquia** |
| --- | --- | --- | --- | --- | --- | --- |
| **N_bufo_Miracatu** | 0.685* |  |  |  |  |  |
| **N_bufo_Cajati** | 1.000 | 0.554* |  |  |  |  |
| **N_bufo_Iguape** | 0.903* | 0.685* | 0.853* |  |  |  |
| **N_bufo_Iporanga** | 1.000* | 0.643* | 1.000 | 0.848* |  |  |
| **N_bufo_Juquia** | 1.000 | 0.630 | 1.000 | 0.589 | 1.000 |  |
| **N_bufo_Cotia** | 0.966* | -0.136 | 0.905 | 0.835* | 0.929 | 0.879 |
