# Supplementary material for: Systematics and phylogeography of the Brazilian Atlantic Forest endemic harvestmen Neosadocus Mello-Leitão, 1926 (Arachnida: Opiliones: Gonyleptidae)
Source: PLoS One. 2021 Jun 2;16(6):e0249746. doi: 10.1371/journal.pone.0249746 (PMC8171921; doi:10.1371/journal.pone.0249746)
Supplement: S12 Table — (DOCX) [file pone.0249746.s017.docx]

**S12 Table.** Pairwise Φ_ST_ values between ***N. bufo*** populations obtained for **ITS2** sequences (*p<0.05).

|  | **N_bufo_Ribeirao_Grande** | **N_bufo_Miracatu** | **N_bufo_Cajati** | **N_bufo_Iguape** | **N_bufo_Iporanga** |
| --- | --- | --- | --- | --- | --- |
| **N_bufo_Miracatu** | 0.678* |  |  |  |  |
| **N_bufo_Cajati** | 0.422 | 0.485 |  |  |  |
| **N_bufo_Iguape** | 0.776* | 0.660* | 0.750 |  |  |
| **N_bufo_Iporanga** | 0.397 | 0.571* | 1.000 | 0.906 |  |
| **N_bufo_Cotia** | 0.812* | 0.268 | 0.667 | 0.847 | 0.833 |
