# Supplementary material for: Systematics and phylogeography of the Brazilian Atlantic Forest endemic harvestmen Neosadocus Mello-Leitão, 1926 (Arachnida: Opiliones: Gonyleptidae)
Source: PLoS One. 2021 Jun 2;16(6):e0249746. doi: 10.1371/journal.pone.0249746 (PMC8171921; doi:10.1371/journal.pone.0249746)
Supplement: S13 Table — (DOCX) [file pone.0249746.s018.docx]

**S13 Table.** Pairwise Φ_ST_ values between ***N. maximus*** populations obtained for **COI** sequences (all values were statistically non-significant).

|  | **N_maximus_Cubatao** | **N_maximus_Santo_Andre** | **N_maximus_Salesopolis** | **N_maximus_Guaruja** |
| --- | --- | --- | --- | --- |
| **N_maximus_Santo_Andre** | 1.000 |  |  |  |
| **N_maximus_Salesopolis** | 1.000 | 1.000 |  |  |
| **N_maximus_Guaruja** | 0.909 | 0.920 | 0.941 |  |
| **N_maximus_Ubatuba** | 0.960 | 0.958 | 0.957 | 0.969 |
