# Supplementary material for: Systematics and phylogeography of the Brazilian Atlantic Forest endemic harvestmen Neosadocus Mello-Leitão, 1926 (Arachnida: Opiliones: Gonyleptidae)
Source: PLoS One. 2021 Jun 2;16(6):e0249746. doi: 10.1371/journal.pone.0249746 (PMC8171921; doi:10.1371/journal.pone.0249746)
Supplement: S15 Table — (DOCX) [file pone.0249746.s020.docx]

**S15 Table.** Pairwise Φ_ST_ values between ***N. robustus*** populations obtained for **COI** sequences (*p<0.05).

|  | **N_robustus_Ribeirao_Grande** | **N_robustus_Cajati** | **N_robustus_Cotia** | **N_robustus_Morretes** | **N_robustus_Guaraquecaba** | **N_robustus_Antonina** | **N_robustus_Cananeia** | **N_robustus_Barra_do_Turvo** | **N_robustus_Ibiuna** | **N_robustus_Guaratuba** | **N_robustus_Faz_Rio_Grande** |
| --- | --- | --- | --- | --- | --- | --- | --- | --- | --- | --- | --- |
| **N_robustus_Cajati** | 0.937* | 0.000 |  |  |  |  |  |  |  |  |  |
| **N_robustus_Cotia** | 0.953 | 0.939 | 0.000 |  |  |  |  |  |  |  |  |
| **N_robustus_Morretes** | 0.835* | 0.824* | 0.797 | 0.000 |  |  |  |  |  |  |  |
| **N_robustus_Guaraquecaba** | 0.813* | 0.802* | 0.746 | 0.252* | 0.000 |  |  |  |  |  |  |
| **N_robustus_Antonina** | 0.994* | 0.986* | 1.000 | -0.032 | 0.363 | 0.000 |  |  |  |  |  |
| **N_robustus_Cananeia** | 0.975 | 0.964 | 1.000 | 0.830 | 0.778 | 1.000 | 0.000 |  |  |  |  |
| **N_robustus_Barra_do_Turvo** | 0.967 | -0.263 | 1.000 | 0.802* | 0.766* | 1.000* | 1.000 | 0.000 |  |  |  |
| **N_robustus_Ibiuna** | 0.946 | 0.931 | 1.000 | 0.780 | 0.726 | 1.000 | 1.000 | 1.000 | 0.000 |  |  |
| **N_robustus_Guaratuba** | 0.969 | 0.956 | 1.000 | 0.770 | 0.713 | 1.000 | 1.000 | 1.000 | 1.000 | 0.000 |  |
| **N_robustus_Faz_Rio_Grande** | 0.971 | 0.959 | 1.000 | 0.800 | 0.752 | 1.000 | 1.000 | 1.000 | 1.000 | 1.000 | 0.000 |
| **N_robustus_Paranagua** | 0.987 | 0.977* | 1.000 | 0.835* | 0.828* | 1.000* | 1.000 | 1.000 | 1.000 | 1.000 | 1.000 |
