# Supplementary material for: Systematics and phylogeography of the Brazilian Atlantic Forest endemic harvestmen Neosadocus Mello-Leitão, 1926 (Arachnida: Opiliones: Gonyleptidae)
Source: PLoS One. 2021 Jun 2;16(6):e0249746. doi: 10.1371/journal.pone.0249746 (PMC8171921; doi:10.1371/journal.pone.0249746)
Supplement: S16 Table — (DOCX) [file pone.0249746.s021.docx]

**S16 Table.** Pairwise Φ_ST_ values between ***N. robustus*** populations obtained for **ITS2** sequences (*p<0.05).

|  | **N_robustus_Ribeirao_Grande** | **N_robustus_Cajati** | **N_robustus_Cotia** | **N_robustus_Morretes** | **N_robustus_Guaraquecaba** | **N_robustus_Antonina** | **N_robustus_Barra_do_Turvo** | **N_robustus_Ibiuna** | **N_robustus_Faz_Rio_Grande** |
| --- | --- | --- | --- | --- | --- | --- | --- | --- | --- |
| **N_robustus_Cajati** | 0.541* |  |  |  |  |  |  |  |  |
| **N_robustus_Cotia** | 0.655* | 0.959* |  |  |  |  |  |  |  |
| **N_robustus_Morretes** | 0.793* | 0.880* | 0.955* |  |  |  |  |  |  |
| **N_robustus_Guaraquecaba** | 0.561* | 0.612* | 0.819* | 0.168* |  |  |  |  |  |
| **N_robustus_Antonina** | 0.729* | 0.800* | 0.921* | -0.011 | 0.101 |  |  |  |  |
| **N_robustus_Barra_do_Turvo** | 0.575* | 0.000 | 1.000* | 0.910* | 0.638* | 0.836* |  |  |  |
| **N_robustus_Ibiuna** | 0.342* | 0.771* | 1.000 | 0.904* | 0.624* | 0.819* | 1.000* |  |  |
| **N_robustus_Faz_Rio_Grande** | 0.597 | 0.920 | 1.000 | 0.333 | -0.409 | 0.143 | 1.000 | 1.000 |  |
| **N_robustus_Paranagua** | 0.669* | 0.934* | 1.000 | 0.464 | -0.007 | 0.342 | 1.000* | 1.000 | 0.000 |
